# Experimental validation of computationally predicted phytoene synthase isoforms encoded by the *Arabidopsis thaliana PSY* gene

**DOI:** 10.1007/s00299-025-03482-1

**Published:** 2025-04-01

**Authors:** Juan Navarro-Carcelen, Manuel Rodriguez-Concepcion

**Affiliations:** Institute for Plant Molecular and Cell Biology (IBMCP), CSIC-Universitat Politècnica de València, 46022 Valencia, Spain

## Abstract

The representative model in the TAIR database of the first and main rate-determining enzyme of the carotenoid pathway, phytoene synthase (PSY), corresponds to a computational prediction artifact.

**Supplementary Information:**

The online version contains supplementary material available at 10.1007/s00299-025-03482-1.

## Introduction

Carotenoids contribute to photosynthesis and photoprotection in green tissues and they function as pigments in non-photosynthetic tissues such as flower petals and ripe fruits. Furthermore, their oxidative cleavage produces bioactive molecules such as abscisic acid and strigolactones. [Rodríguez-Concepción et al. 2018]. The first committed step of the carotenoid pathway is the condensation of two molecules of geranylgeranyl diphosphate (GGPP) to form phytoene in a reaction catalyzed by the enzyme phytoene synthase (PSY). As the main-rate-determining enzyme of the carotenoid pathway, PSY is usually encoded by small gene families in plants [Zhou et al. 2022]. By contrast, a single *PSY* gene is found in *Arabidopsis thaliana*, a plant that does not naturally overaccumulate carotenoids in non-photosynthetic organs. The TAIR database indicates that the Arabidopsis *PSY* gene (*At5g17230*) can potentially produce four different transcripts and two different proteins (https://www.arabidopsis.org/locus?key=133705). Here we tested whether the two proteins potentially resulting from the single Arabidopsis *PSY* gene are true PSY enzymes.

## Results and discussion

**Fig. 1:**
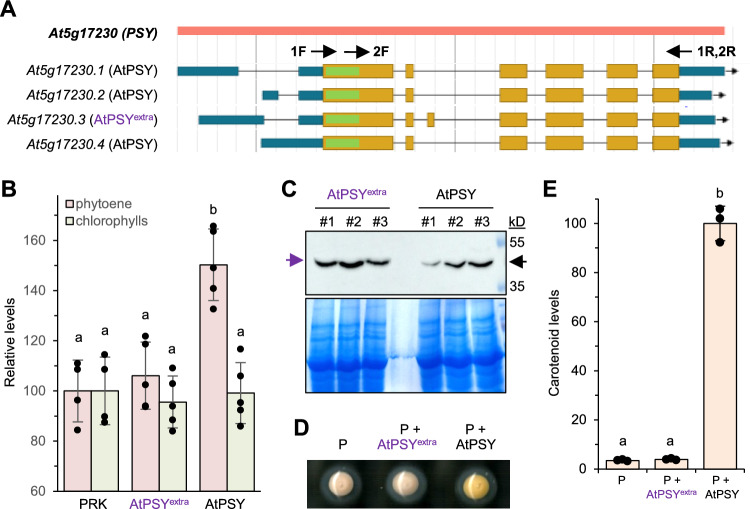
Experimental validation of predicted variants encoded by the Arabidopsis *PSY* gene. **A** *At5g17230* (*PSY*) gene model predictions in the TAIR database. Exons for UTRs and CDS are represented as blue and orange boxes, respectively, and introns are marked as lines. The plastid-targeting sequence is marked in green. The position of primers used for the cloning of cDNA sequences is indicated. **B** Quantification of phytoene and chlorophyll levels in NFZ-treated *N. benthamiana* leaves agroinfiltrated with constructs to express HA-tagged versions of AtPSY or AtPSY^extra^. A PRK-myc construct was used as a control. Columns and error bars correspond to mean ± SD values represented relative to levels in control leaves. **C** Immunoblot analysis of HA-tagged AtPSY or AtPSY^extra^ proteins accumulated in three independent replicates of the samples described in (B). Size of protein standards (in kD) is indicated on the right, and arrows mark the position of AtPSY or AtPSY^extra^ proteins. The lower picture shows the Coomassie staining of the gel prior to blotting. **D** Cell pellets of *E. coli* strains transformed with pAC-85b alone (P) or together with pET32-AtPSY^extra^ or pET32-AtPSY. **E** Carotenoid levels produced by strains described in (**D**). Levels were normalized to optical density at 600 nm and are represented relative to those in pET32-AtPSY samples. In (**B**) and (**E**) plots, different letters represent statistically significant differences (*p* < 0.05) among means according to one-way ANOVA followed by post hoc Tukey’s tests

The four different transcripts presumably produced by the Arabidopsis *PSY* gene are named as gene models *At5g17230.1* to *At5g17230.4* (Fig. 1A). From them, only *At5g17230.3* differs in the protein-coding region, showing an extra exon. The protein encoded by the *At5g17230.3* model (herein referred to as AtPSY^extra^) contains an extra sequence of 15 residues (VMLKVDFYKQSIVAL) that is not present in the protein predicted by the other three Arabidopsis gene models (referred to as AtPSY) or in any of the three tomato PSY paralogs (Supplementary Figure S1). The extra sequence in the AtPSY^extra^ variant has only been detected in a few expressed sequence tags (ESTs) [Campbell et al. 2014] and peptides [Castellana et al. 2008]. Despite the absence of solid experimental evidence supporting AtPSY^extra^ natural occurrence or activity, *At5g17230.3* is currently selected as a representative gene model in the TAIR database (https://www.arabidopsis.org/locus?key=133705).

To confirm whether the two Arabidopsis protein sequences predicted by the TAIR gene models, i.e., AtPSY (predicted by *At5g17230.1*, *At5g17230.2* and *At5g17230.4*) and AtPSY^extra^ (from *At5g17230.3*) are true PSY enzymes, we compared their in vivo activity in plant and bacterial systems. PCR primers annealing on the regions harboring the translation start and end codons (Fig. 1A and Supplementary Table S1) failed to amplify the sequence encoding AtPSY^extra^ from different Arabidopsis cDNA sources, including leaves and flowers. We therefore used a synthetic cDNA sequence encoding this predicted isoform (Fig. 1A). To analyze the activity of the two PSY variants in living plant cells, the production of phytoene (the direct product of PSY activity) was quantified in *Nicotiana benthamiana* leaves agroinfiltrated with constructs to express either natural (AtPSY) or synthetic (AtPSY^extra^) cDNA sequences fused to a C-terminal hemagglutinin (HA) tag under the control of the constitutive *35S* promoter. A similar construct expressing the Arabidopsis enzyme phosphoribulokinase (PRK) was used as a control lacking PSY activity [Barja et al. 2021]. To prevent conversion of phytoene into downstream carotenoids by the endogenous carotenoid pathway, agroinfiltrated leaves were further treated with norflurazon (NFZ), an inhibitor that prevents phytoene desaturation [Iglesias-Sanchez et al. 2024]. Levels of chlorophyll, a chloroplast metabolite that should not be affected by PSY activity, were similar in all samples (Fig. 1B). By contrast, leaves agroinfiltrated with AtPSY constructs showed significantly higher amounts of phytoene than AtPSY^extra^ samples and PRK controls (Fig. 1B). This result suggests that AtPSY^extra^ lacks PSY activity, as similar levels of phytoene detected in AtPSY^extra^ and PRK leaves derive from the activity of endogenous *N. benthamiana* PSY enzymes. Aliquots of the same samples used for metabolite analyses were used for protein extraction and immunoblot analysis with anti-HA antibodies. The results showed that the higher phytoene levels detected in AtPSY samples were not the result of increased protein accumulation compared to AtPSY^extra^ leaves (Fig. 1C). Furthermore, the presence of AtPSY^extra^ proteins at detectable levels indicates that the failure to produce more phytoene than the PRK control (Fig. 1B) is likely due to the absence of detectable PSY activity.

To confirm whether AtPSY^extra^ lacked PSY activity, we next used *Escherichia coli* cells transformed with pAC-85b, a plasmid with a synthetic operon that only lacks a PSY-encoding gene to produce the orange carotenoid β-carotene from endogenous bacterial precursors [Iglesias-Sanchez et al. 2024]. Versions of AtPSY and AtPSY^extra^ lacking the predicted N-terminal plastid targeting peptide were cloned into pET32 vectors for bacterial expression. Cultures carrying pAC-85b and pET32-AtPSY showed a characteristic orange color whereas those co-transformed with pAC-85b and pET32-AtPSY^extra^ were undistinguishable from controls transformed only with pAC-85b (Fig. 1D). In agreement with the color phenotype, only cells harboring pET32-AtPSY were found to produce β-carotene (Fig. 1E). Together, our results indicate that AtPSY ^extra^ lacks detectable enzymatic activity, likely because it corresponds to a computational prediction artifact.

The reported results are a good example that despite advancements in high-throughput sequencing, proteomics, and bioinformatic algorithms to predict coding sequences, experimental validation is a must to ascertain the biological relevance of the predictions. Such experimental data should next feed improved computational models for enhancing our ability to accurately predict gene isoform diversity, which has important implications for understanding gene function and regulation in various biological contexts.

## Materials and methods

*Gene constructs*: See Supplementary Methods.

### Expression in plants and bacteria

Leaves from six-week-old *Nicotiana benthamiana* plants were agroinfiltrated as described (Morelli et al. 2023) and one day later treated with 2 μM norflurazon (NFZ) as described [Iglesias-Sanchez et al. 2024]. Samples of leaves from 4 to 6 different plants were collected 4 days after agroinfiltration and snap-frozen in liquid nitrogen before lyophilization and grinding to a fine powder of dry tissue. *Escherichia coli* TOP10 cells were transformed with plasmid pAC-85b and pET32 constructs, and positive transformants were selected on Luria broth (LB) solid medium supplemented with antibiotics. Overnight-grown 5 ml precultures of at least three colonies of each combination were used to quantify carotenoid production as described [Iglesias-Sanchez et al. 2024].

*Immunoblot and metabolite assays*: See Supplementary Methods.

## Supplementary Information

Below is the link to the electronic supplementary material.

Supplementary file1 (DOCX 2038 KB)

## Data Availability

All data generated during this study are included in this published article and its supplementary information file.
