# Supplementary material for: Experimental validation of computationally predicted phytoene synthase isoforms encoded by the Arabidopsis thaliana PSY gene
Source: Plant Cell Rep. 2025 Apr 1;44(4):93. doi: 10.1007/s00299-025-03482-1 (PMC11961509; doi:10.1007/s00299-025-03482-1)
Supplement: Supplementary file 1 — Supplementary file1 (DOCX 2038 KB) [file 299_2025_3482_MOESM1_ESM.docx]

**Supplementary Information**

**Experimental validation of computationally-predicted phytoene synthase isoforms encoded by the *Arabidopsis thaliana PSY* gene**

Juan NAVARRO-CARCELEN and Manuel RODRIGUEZ-CONCEPCION*

**Supplementary Methods**

**Gene constructs.**

The full coding region of the Arabidopsis *PSY* cDNA was PCR-amplified from leaf tissues using primers AtPSY-attB1-F and AtPSY-attB2-R (Supplementary Table S1) and cloned into pGWB414 to create the AtPSY-HA fusion as described [Iglesias-Sanchez et al., 2024]. The sequence encoding AtPSY^extra^ (Supplementary Figure S2) was synthetically generated as a gBlock (IDT) and then used to create the HA-fused version as described for AtPSY. The construct encoding PRK-myc and plasmid pAC-85b were previously available in the lab [Barja et al., 2021; Iglesias-Sanchez et al., 2024]. For experiments in bacteria, cDNA sequences lacking the plastid-targeting motif were PCR-amplified using primers cAtPSY-attB1-F and cAtPSY-attB2-R (Supplementary Table S1) and cloned into pDONR207 and subsequently pET32GW using Gateway technology.

**Immunoblot assays.**

Plant protein extracts were prepared from three independent *N. benthamiana* leaves agroinfiltrated with the appropriate constructs and used for immunoblot analysis with commercial HA-HRP antibodies (Invitrogen) as described [Morelli et al., 2023].

**Metabolite analysis.**

Carotenoids and chlorophylls were extracted from plant tissues, separated, identified and quantified by HPLC-DAD [Barja et al., 2021]. Phytoene was detected at 280 nm and chlorophylls at 650 nm. Carotenoids were extracted from bacteria using acetone and used for spectrophotometric quantification at 472 nm as described [Iglesias-Sanchez et al., 2024].

**Supplementary Figures**

 **Figure S1. Aligment of predicted PSY protein sequences from Arabidopsis and tomato.** Alignment was carried out using the Uniprot Align platform with default parameters. The predicted N-terminal plastid-targeting sequence in the Arabidopsis proteins is boxed in green, and the extra sequence present only in the predicted AtPSY^extra^ protein is boxed in purple. Sequence data can be found under the following accession numbers: At5g17230 (AtPSY and AtPSY^extra^), Solyc03g031860 (SlPSY1), Solyc02g081330 (SlPSY2), and Solyc01g005940 (SlPSY3).

**atgtcttcttctgtagcagtgttatgggttgctacttcttctctaaatccagacccaatgaacaattgtgggttggtaagggttctagaatcttctagactgttctctccttgtcagaatcagagactaaacaaaggtaagaagaagcagataccaacttggagttcttcttttgtaaggaaccgaagtagaagaattggtgttgtgtct**tcaagcttagtagcaagtccttctggagagatagctctttcatctgaagagaaggtttacaatgttgtgttgaaacaagctgctttggtgaacaaacagctaaggtcttcttcttatgaccttgatgtgaagaaaccacaagatgttgttcttcctgggagtttgagtttgttgggtgaagcttatgatcgatgcggtgaagtttgcgctgaatatgctaagacgttttatcttggaactttgcttatgacacccgaaaggcgaaaggcgatttgggcaatctac**gtgatgctcaaagtagatttctacaaacaatctattgtggctctt**gtttggtgtagaagaactgatgaacttgtggatgggccaaatgcttcacatataactcccatggctttagatagatgggaagcaaggttagaagatcttttccgtggtcgtcctttcgatatgcttgatgctgctctcgctgatacagttgctagatacccggtcgatattcagccatttcgagacatgatcgaaggaatgagaatggacttgaagaaatcgagataccagaacttcgatgatctatacctttactgctactacgtcgctggaaccgtcggattgatgagcgttccggttatgggaatcgatcctaagtcgaaagcaacaaccgaaagtgtttacaacgctgccttggcccttggtatagccaatcagcttactaacatactcagagacgtaggcgaagatgcgagaagaggaagggtttatctgcctcaggatgaattggctcaggctggtctttcagatgaagacatattcgccggaaaagtaactgataaatggagaaacttcatgaaaatgcagcttaaacgagcaagaatgttcttcgacgaagctgagaaaggcgtcaccgagctcagtgccgctagcagatggcctgtatgggcttcattgctattgtacaggagaatactggacgagattgaagcgaatgattacaacaattttactaagagagcttatgtggggaaagtcaagaaaattgcagctttgccattggcttatgctaaatcagtactaaagacttcaagttcaagactatcgatatga

**Figure S2. Sequence of the synthetic cDNA encoding the AtPSY+ protein.** The sequences encoding the predicted N-terminal plastid-targeting and the extra domain absent in AtPSY are marked in green and purple, respectively.

**Supplementary Tables**

**Table S1. Primers used in this work.**

| **Code Fig. 1** | **Name** | **Sequence (5'-3')** |
| --- | --- | --- |
| 1F | AtPSY-attB1-F | GGGGACAAGTTTGTACAAAAAAGCAGGCTGGATGTCTTCTTCTGTAGCAGTG |
| 1R | AtPSY-attB2-R | GGGGACCACTTTGTACAAGAAAGCTGGGTCTATCGATAGTCTTGAACTTGAAG |
| 2F | cAtPSY-attB1-F | GGGGACAAGTTTGTACAAAAAAGCAGGCTTCATGTCAAGCTTAGTAGCAAGTCC |
| 2R | cAtPSY-attB2-R | GGGGACCACTTTGTACAAGAAAGCTGGGTTTCATATCGATAGTCTTGAACTTG |
